# Comprehensive Interactome Analysis Reveals that STT3B Is Required for N-Glycosylation of Lassa Virus Glycoprotein

**DOI:** 10.1128/JVI.01443-19

**Published:** 2019-11-13

**Authors:** Shenglin Zhu, Weiwei Wan, Yanjun Zhang, Weijuan Shang, Xiaoyan Pan, Lei-Ke Zhang, Gengfu Xiao

**Affiliations:** a State Key Laboratory of Virology, Wuhan Institute of Virology, Chinese Academy of Sciences, Wuhan, China; b University of Chinese Academy of Sciences, Beijing, China; University of Kentucky College of Medicine

**Keywords:** Lassa virus, glycoprotein, interactome, STT3B, NGI-1

## Abstract

Lassa virus (LASV) is the causative agent of a fatal hemorrhagic fever in humans. The glycoprotein (GP) of LASV mediates viral entry into host cells, and correct processing and modification of GP by host factors is a prerequisite for virus replication. Here, using an affinity purification-coupled mass spectrometry (AP-MS) strategy, 591 host proteins were identified as interactors of LASV GP. Gene ontology analysis was performed to functionally annotate these proteins, and the oligosaccharyltransferase (OST) complex was highly enriched. Functional studies conducted by using CRISPR-Cas9-mediated knockouts showed that STT3A and STT3B, the two catalytically active isoforms of the OST complex, are essential for the propagation of the recombinant arenavirus rLCMV/LASV glycoprotein precursor, mainly via affecting virus infectivity. Knockout of *STT3B*, but not *STT3A*, caused hypoglycosylation of LASV GP, indicating a preferential requirement of LASV for the STT3B-OST isoform. Furthermore, double knockout of magnesium transporter 1 (*MAGT1*) and tumor suppressor candidate 3 (*TUSC3*), two specific subunits of STT3B-OST, also caused hypoglycosylation of LASV GP and affected virus propagation. Site-directed mutagenesis analysis revealed that the oxidoreductase CXXC active-site motif of MAGT1 or TUSC3 is essential for the glycosylation of LASV GP. NGI-1, a small-molecule OST inhibitor, can effectively reduce virus infectivity without affecting cell viability. The STT3B-dependent N-glycosylation of GP is conserved among other arenaviruses, including both the Old World and New World groups. Our study provided a systematic view of LASV GP-host interactions and revealed the preferential requirement of STT3B for LASV GP N-glycosylation.

**IMPORTANCE** Glycoproteins play vital roles in the arenavirus life cycle by facilitating virus entry and participating in the virus budding process. N-glycosylation of GPs is responsible for their proper functioning; however, little is known about the host factors on which the virus depends for this process. In this study, a comprehensive LASV GP interactome was characterized, and further study revealed that STT3B-dependent N-glycosylation was preferentially required by arenavirus GPs and critical for virus infectivity. The two specific thioredoxin subunits of STT3B-OST MAGT1 and TUSC3 were found to be essential for the N-glycosylation of viral GP. NGI-1, a small-molecule inhibitor of OST, also showed a robust inhibitory effect on arenavirus. Our study provides new insights into LASV GP-host interactions and extends the potential targets for the development of novel therapeutics against Lassa fever in the future.

## Abstract (precis)

Glycoproteins play vital roles in the arenavirus life cycle by facilitating virus entry and participating in the virus budding process. N-glycosylation of GPs is responsible for their proper functioning; however, little is known about the host factors on which the virus depends for this process. In this study, a comprehensive LASV GP interactome was characterized, and further study revealed that STT3B-dependent N-glycosylation was preferentially required by arenavirus GPs and critical for virus infectivity. The two specific thioredoxin subunits of STT3B-OST MAGT1 and TUSC3 were found to be essential for the N-glycosylation of viral GP. NGI-1, a small-molecule inhibitor of OST, also showed a robust inhibitory effect on arenavirus. Our study provides new insights into LASV GP-host interactions and extends the potential targets for the development of novel therapeutics against Lassa fever in the future.

## INTRODUCTION

Lassa virus (LASV) is the causative agent of Lassa fever, which is estimated to cause 100,000 to 300,000 infections annually in west Africa, with approximately 5,000 deaths (1). Nosocomial epidemics of Lassa fever are associated with higher case fatality rates up to 36 to 65% (2, 3). A seasonal outbreak of Lassa fever occurred in Nigeria in 2018 and led to more than 100 deaths, reemphasizing the threat of LASV on public health (4). There is no U.S Food and Drug Administration-approved vaccine for LASV, and the only treatment option for Lassa fever is the nucleoside analog drug ribavirin, which is most effective when given in the early stage of the disease (5). Due to its high morbidity and mortality rates, LASV is classified as a biosafety level 4 (BSL-4) agent (6) and a category A priority pathogen (7).

LASV belongs to the *Mammarenavirus* genus (member of the *Arenaviridae* family) (8), a genus of enveloped, negative-strand RNA viruses that can be divided into two groups: Old World (OW) and New World (NW) (9). The OW family of arenaviruses includes LASV and lymphocytic choriomeningitis virus (LCMV), a neglected human pathogen distributed worldwide (10, 11), while the NW family of arenaviruses includes Junín virus (JUNV) and Machupo virus (MACV), the causative agents of Argentine hemorrhagic fever and Bolivian hemorrhagic fever, respectively (9).

The glycoprotein (GP) of LASV forms spikes on the surface of the virion and is the sole antigen responsible for eliciting a virus-neutralizing antibody response (12). The LASV GP is synthesized as a single glycoprotein precursor (GPC) and is subsequently cleaved into three segments by host signal peptidase (13) and subtilisin-kexin-isozyme-1/site 1 protease (SKI-1/S1P) (14–16), producing a stable signal peptide (SSP), a receptor binding subunit (GP1), and a class I membrane fusion subunit (GP2), which together form a trimer of GP heterotrimers on the virion surface through noncovalent interactions (17–21). GPs of NW arenaviruses, such as JUNV and MACV, bind to transferrin receptor 1 (TfR1) to facilitate entry (22), while OW arenaviruses use alpha-dystroglycan (α-DG) or neuropilin-2 (NRP2) as their cell surface receptors (23, 24). Upon delivery to the late endosome, the GP of LASV undergoes an acidic pH-induced receptor switch to the intracellular receptor LAMP1, thereby facilitating membrane fusion to release the viral ribonucleoprotein complex into the cytosol (25). Arenavirus GPs also play an important role in the budding of virus progeny by interacting with Z protein, which provides the driving force of budding (26). By recruiting the matrix protein Z and the assembled nucleocapsid to virus budding sites, GP determines the apical release of nascent LASV from polarized epithelial cells (27).

Asparagine-linked (N-)glycosylation plays an important role in the folding, stabilization, oligomerization, quality control, sorting, and transport of GPs (28, 29), and all of these processes are essential for maintaining normal viral GP functions. For example, glycosylation of influenza hemagglutinin (HA) directs the proper folding and trafficking of nascent polypeptides (30) and thus modulates the viral binding ability and regulates viral release (31). Loss of glycans in HIV-1 gp120 significantly reduces virus binding to CD4 (32). LASV GP possesses 11 potential sites for N-glycan modification, 7 of which are necessary for its proteolytic cleavage (33). Despite the lack of cleavage in these latter glycosylation mutants, transport of a single glycosylation site-mutated GPC to the cell surface is not impaired, indicating that a single N-glycosylation site and proteolytic cleavage are not necessary for its intracellular trafficking (33). N-glycans in LASV GP also promote immune evasion in humans by shielding the virus from host neutralizing antibodies (34). This finding is in line with historical reports that passive serum therapy provided to cynomolgus monkeys or patients generated only limited protection efficacy (35, 36).

Novel therapeutic strategies to control arenavirus-induced diseases require a thorough understanding of virus-host interactions. However, little is known about the comprehensive host interactome of LASV GP. In this study, an affinity purification-coupled mass spectrometry (AP-MS) strategy was used to identify host proteins interacting with LASV GP, and the oligosaccharyltransferase (OST) complex was highlighted. The OST complex is responsible for the N-glycosylation process in the endoplasmic reticulum (ER) lumen, where it catalytically transfers a preassembled oligosaccharide to the amide group of an asparagine residue within the consensus N-X-T/S motif of a nascent polypeptide (37). Multiple subunits of the mammalian OST complex have been reported, including defender against cell death 1 (DAD1), OST48, OST4, OSTC, ribophorin I (RPN1), ribophorin II (RPN2), TUSC3, MAGT1, STT3A, and STT3B (38–40). The STT3 protein is the central enzyme of the OST complex, and two isoforms exist in mammalian cells: STT3A and STT3B (37). MAGT1 and TUSC3 are specific subunits of STT3B with overlapping functions. Either MAGT1 or TUSC3 is incorporated into STT3B complexes and endows STT3B with the capability to posttranslationally glycosylate cysteine-proximal acceptor sites via their oxidoreductase activity (41). Functional studies showed that STT3A and STT3B are essential for the propagation of a recombinant arenavirus rLCMV/LASV GPC. Although most glycoproteins in host cells are efficiently modified by STT3A in a cotranslational manner (42), our study indicated that LASV GP was preferentially modified by the STT3B-OST isoform. Our study provides new insights into the host interactome of LASV GP and extends the potential targets for the development of novel therapeutics in the future.

## RESULTS

### Identification of host proteins interacting with LASV GP.

**FIG 1:**
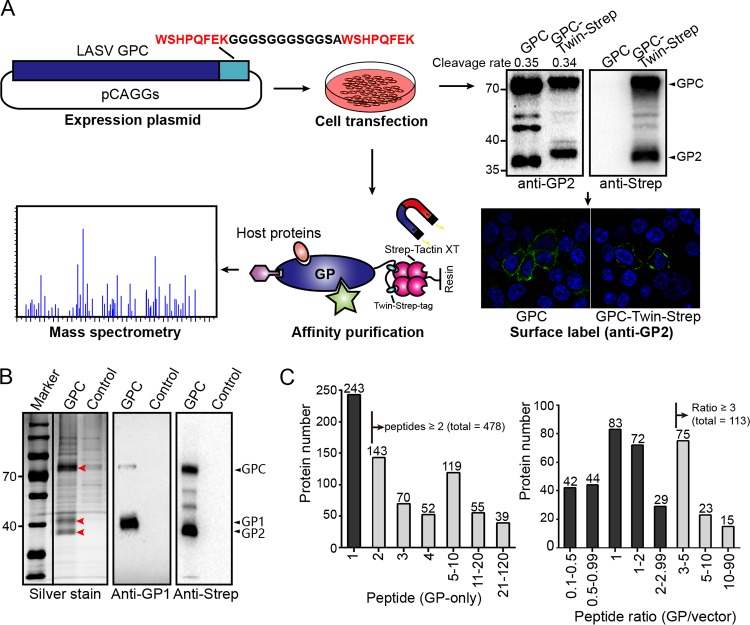
Identification of host proteins interacting with LASV GP. (A) Schematic diagram of the experimental strategy used to identify host proteins interacting with LASV GP. HEK293T cells were transfected with a pCAGGs plasmid encoding LASV GP with a C-terminal Twin-Strep-tag or a pCAGGs plasmid expressing only the Twin-Strep-tag as a control. At 48 h posttransfection, the cells were harvested and lysed by using a mild nonionic detergent buffer at 4°C. LASV GP-associated host interactors were pulled down from cell lysates by magnetic Sepharose beads coated with Strep-Tactin XT. Purified proteins were digested into peptides by trypsin and subjected to liquid chromatography-tandem mass spectrometry analysis. Western blotting and immunofluorescence were used to test whether the Twin-Strep-tag would affect the proteolytic cleavage, glycosylation, and cell surface transport of LASV GP. The cleavage rates of tagged and untagged GP2 are shown above, as determined by densitometric quantification. (B) Silver-stained SDS-PAGE gel of purified LASV GP together with host interactors or the control group. Western blot analysis was also conducted to show the bands of viral GP1 and GP2, as detected by GP1-specific serum and an anti-Strep antibody, respectively. The silver-stained and Western blot gels shown here are representatives of three independent replicates. (C) The left column diagram shows the distribution of detected peptide counts from proteins identified in only the GP group and not in the control group. The right column diagram shows the distribution of peptide count ratios from proteins identified in both the GP group and the control group (GP/control). Proteins that fulfilled our filtration standard in the two groups were considered to be valid interactors.

To identify the host proteins interacting with LASV GP, we adopted a strategy to tag its C terminus with a Twin-Strep-tag, which is advantageous for the one-step purification of protein complexes from cell lysates (43–45). No significant changes of the proteolytic cleavage, glycosylation, or cell surface transport were observed between the tagged and untagged LASV GPs (Fig. 1A). Then, plasmids encoding the tagged LASV GP or vector plasmid as a control were transfected into HEK293T cells. At 48 h posttransfection, the cells were harvested, and LASV GP-associated host proteins were purified from the cell lysates by magnetic Sepharose beads coated with Strep-Tactin XT and subjected to Western blot analysis using a GP1-specific serum or anti-Strep antibody. Cleaved GP1 and GP2 were purified simultaneously from the cell lysates, together with a noncleaved form of GPC (Fig. 1B). The retention of cleaved GP1 with purified GP2 suggests that viral GP remained in the prefusion conformation during the purification process since GP1 and GP2 interact with each other through unstable noncovalent interactions, and irreversible conformation changes during the membrane fusion process lead to their disassociation (20, 25). Then, eluted proteins were trypsin digested and subjected to mass spectrometry (MS) analysis. Three independent replicate experiments were performed, and a total of 1,104 host proteins were identified from at least one independent replicate (see Table S1 in the supplemental material). Only proteins meeting the following criteria were considered as interactors of GP: (i) for proteins identified in only the GP group but not the control group, at least two peptides should be identified, or (ii) for proteins identified in both the GP and control groups, the GP versus control group peptide count ratios should be >3 (Fig. 1C). With this standard, 591 proteins were considered as valid interactors of LASV GPs. α-DG, the host receptor of OW arenaviruses (23), was detected in the GP groups with high abundance but not in the control groups (Table S1).

### Gene ontology analysis of LASV GP-interacting proteins highlighted the OST complex.

**FIG 2:**
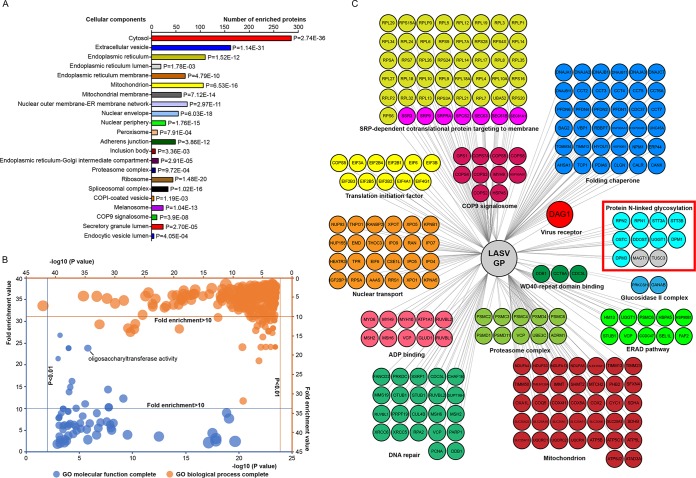
Gene ontology (GO) analysis of LASV GP-interacting proteins highlighted the OST complex. (A) Cellular component distribution of LASV GP interactors as determined in PANTHER. A total of 591 proteins were clustered into 22 groups, demonstrating the extensive cellular distribution of LASV GP interactors. The raw *P* values determined by the Fisher exact test are presented beside the column. (B) GO overrepresentation analysis based on molecular function and biological process annotations. Subsets of generated terms with ranked fold-enrichment values and –log_10_(*P* values) are visualized in the scatterplot graph. The bubble sizes are related to the number of mapped proteins. Redundant GO terms were first eliminated by REVIGO based on semantic similarity and further summarized manually. The top representative functional subsets with high fold enrichment values (>10) are highlighted in the scatterplot graph (central region) and listed in Tables 1 and 2. The test was performed using the Fisher exact test with an FDR multiple test correction of <0.05. (C) Representative overview of the LASV GP interactome classified based on both biological processes and molecular functions, which were visualized by Cytoscape. Subunits of the OST complex are highlighted by a red square. MAGT1 and TUSC3 were validated by the coimmunoprecipitation assay shown below and are also presented here. Note that not all the interactors are presented here. In addition, note that one protein may be classified into more than one subset.

To functionally annotate interactors, 591 host proteins identified here were submitted to Protein Annotation Through Evolutionary Relationship (PANTHER) to perform gene ontology (GO) analysis (46). As shown in Fig. 2A and Table S1 in the supplemental material, 591 proteins were clustered into 22 groups based on the cellular components, demonstrating the extensive cellular distribution of LASV GP interactors. Multiple proteins were clustered into compartments of the host secretory system, such as the ER, ER-Golgi intermediate compartment, Golgi apparatus, and plasma membrane. Interactors residing in these cellular components might participate in the maturation process of LASV GP and thus affect viral replication.

To further explore the biological significance of GP-associated interactors, statistical overrepresentation tests for GO molecular functions and biological processes were also performed. Redundant GO terms were first eliminated by REVIGO based on semantic similarity (47) and further summarized manually. Functional subsets of proteins with ranked fold enrichment values and –log10(*P* values) are shown in Fig. 2B. The top overrepresented functional subsets from GO molecular function and biological process analyses with high fold enrichment values (>10) are highlighted in the scatterplot graph (central region) and listed in Tables 1 and 2. Proteins responsible for host OST activity were highly overrepresented. STT3A and STT3B, the two isoforms of the catalytic subunit of the mammalian OST complex, together with their auxiliary subunits were identified as GP interactors here (Fig. 2C). In addition, proteins involved in the protein folding process and proteasome activity were also significantly enriched in the data set, hinting at possible roles of these proteins in arenavirus GP physiology. Interestingly, multiple proteins responsible for nuclear transport or Ran GTPase binding were also enriched in the data set, consistent with the results of a proteomic survey of JUNV virions (48), while their roles in virus replication remain unknown.

**TABLE 1:** Top overrepresented functional subsets from GO molecular function analyses with high fold-enrichment values

Rank	Term_ID	Description	Fold enrichment
1	GO:0071987	WD40-repeat domain binding	26.83
2	GO:0004579	Dolichyl-diphosphooligosaccharide-protein glycotransferase activity	23.85
3	GO:0015288	Porin activity	23.85
4	GO:0036402	Proteasome-activating ATPase activity	23.85
5	GO:0036033	Mediator complex binding	21.46
6	GO:0001094	TFIID-class transcription factor complex binding	16.26
7	GO:0030898	Actin-dependent ATPase activity	14.9
8	GO:0048027	mRNA 5′-UTR binding	14.31
9	GO:0002161	Aminoacyl-tRNA editing activity	13.76
10	GO:0008536	Ran GTPase binding	12.24
11	GO:0030515	snoRNA binding	11.01
12	GO:0016875	Ligase activity, forming carbon-oxygen bonds	10.22
13	GO:0004812	Aminoacyl-tRNA ligase activity	10.22
14	GO:0008139	Nuclear localization sequence binding	10.02
15	GO:0140142	Nucleocytoplasmic carrier activity	9.94

**TABLE 2:** Top overrepresented functional subsets from GO biological process analyses with high fold enrichment values

Rank	Term_ID	Description	Fold enrichment
1	GO:1904869	Regulation of protein localization to Cajal body	31.79
2	GO:0000338	Protein deneddylation	21.46
3	GO:1901503	Ether biosynthetic process	17.88
4	GO:0035964	COPI-coated vesicle budding	17.88
5	GO:0008611	Ether lipid biosynthetic process	17.88
6	GO:0046504	Glycerol ether biosynthetic process	17.88
7	GO:0006610	Ribosomal protein import into nucleus	17.88
8	GO:0034723	DNA replication-dependent nucleosome organization	17.88
9	GO:0000183	Chromatin silencing at ribosomal DNA	17.88
10	GO:0000470	Maturation of LSU-rRNA	17.88
11	GO:0045898	Regulation of RNA polymerase II transcriptional preinitiation complex assembly	15.33
12	GO:0007084	Mitotic nuclear envelope reassembly	14.31
13	GO:0006614	SRP-dependent cotranslational protein targeting to membrane	14.01
14	GO:0106074	Aminoacyl-tRNA metabolism involved in translational fidelity	13.76
15	GO:0000463	Maturation of LSU-rRNA from tricistronic rRNA transcript (SSU-rRNA, 5.8S rRNA, and LSU-rRNA)	13.76
16	GO:0048025	Negative regulation of mRNA splicing, via spliceosome	13.63
17	GO:0006613	Cotranslational protein targeting to membrane	13.33
18	GO:0042273	Ribosomal large subunit biogenesis	13.23
19	GO:0045039	Protein insertion into mitochondrial inner membrane	13.01
20	GO:0034982	Mitochondrial protein processing	13.01
21	GO:0045047	Protein targeting to the ER	13.01
22	GO:0090151	Establishment of protein localization to mitochondrial membrane	12.62
23	GO:0000715	Nucleotide-excision repair, DNA damage recognition	12.44
24	GO:0006413	Translational initiation	12
25	GO:0042776	Mitochondrial ATP synthesis coupled proton transport	11.92

**FIG 3:**
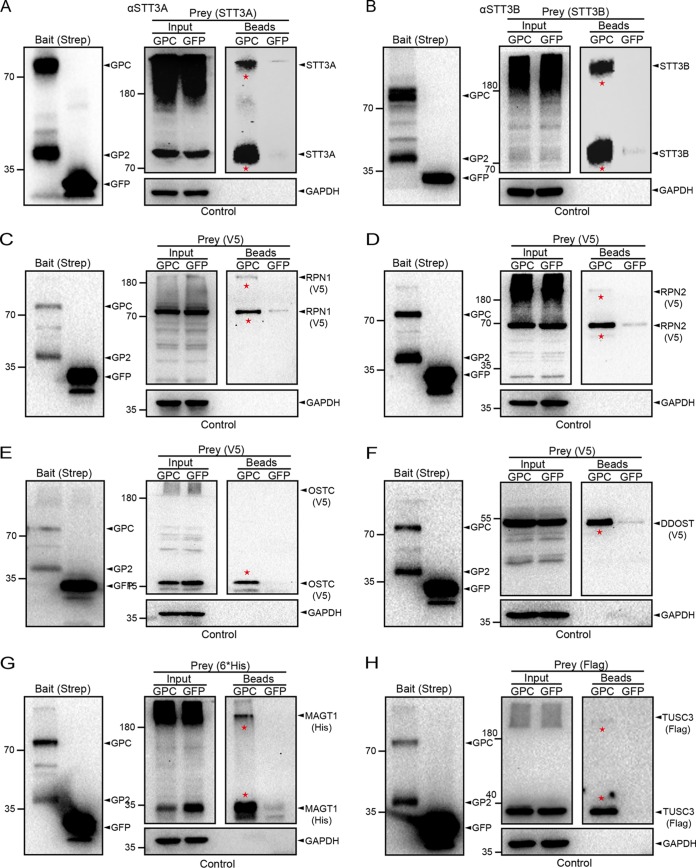
Confirmation of the interactions between LASV GP and subunits of the OST complex by coimmunoprecipitation and Western blot analyses. HEK293T cells were transfected with a pCAGGs plasmid encoding Twin-Strep-tagged LASV GP or GFP, and at 48 h posttransfection the cells were harvested and lysed at 4°C. LASV GP and GFP were pulled down from the cell lysates by magnetic Sepharose beads coated with Strep-Tactin XT as bait (Input). Purified bead fractions were screened for endogenous STT3A (A) and STT3B (B) by SDS-PAGE and Western blot analysis using antibodies specific to human STT3A and STT3B, respectively. (C to H) A pCAGGs plasmid encoding V5-tagged RPN1, RPN2, OSTC, or DDOST; 6×His-tagged MAGT1; or Flag-tagged TUSC3 was cotransfected into HEK293T cells with a plasmid expressing LASV GP or GFP. Purified bead fractions were screened for RPN1 (C), RPN2 (D), OSTC (E), DDOST (F), MAGT1 (G), and TUSC3 (H) by SDS-PAGE and Western blot analysis with antibodies specific to the V5 (RPN1, RPN2, OSTC, and DDOST), 6×His (MAGT1) or Flag (TUSC3) epitopes. LASV GP and GFP were detected by an anti-Strep antibody (A to H, left). GAPDH was also detected as a control of cell lysate input and bead fraction output (A to H, bottom). Bands indicating specific interactions between LASV GP and the OST subunits are marked with a red star. Note that some of the OST subunits were found to migrated into diffused bands and formed high-molecular-weight aggregates in the SDS-PAGE gels. LASV GP but not the control GFP was able to pull down all of the subunits identified in our MS data set.

To verify the interactions between LASV GP and the OST subunits identified here, coimmunoprecipitation and Western blot analyses were performed. Twin-Strep-tagged LASV GP with its associated proteins was purified from cell lysates. Green fluorescent protein (GFP) tagged with a Twin-Strep-tag was also set as the control bait. Endogenous STT3A and STT3B or exogenously expressed RPN1, RPN2, OSTC, and DDOST/OST48 were detected in the GP fraction but not in the GFP-purified fraction (Fig. 3A to F). Most of the OST subunits (excluding DDOST) migrated into diffused bands and formed high-molecular-weight aggregates, probably because of stable associations with other subunits of the OST complex. We also compared our data set to a previous interactome of LCMV GP and found that 127 proteins were common (Table S1) (49). Interestingly, subunits of the OST complex were also identified in the interactome of LCMV GP, including STT3B, DDOST, RPN1, RPN2, and MAGT1. These results suggested a high confidence level of our interactome results and confirmed that LASV GP interacted specifically with the host OST complex.

### CRISPR-Cas9-mediated isoform-specific knockouts of the OST complex impaired the propagation of the rLCMV/LASV GPC virus.

The GPs of arenaviruses are highly glycosylated (34), and the absence of even a single GP glycosylation site could globally attenuate the arenavirus *in vivo* (50). We hypothesized that the OST complex is central to GP modification and might therefore play a vital role in virus propagation. Because of partly redundant functions of the two isoforms in N-linked glycosylation, most glycoproteins in host cells can be modified by both OST isoforms except for a few special proteins that are preferentially modified by either STT3A or STT3B (51). STT3A and STT3B also display distinct enzymatic properties, and differential utilization of the two isoforms may represent distinct glycosylation patterns (52). It remains unknown whether the glycosylation of LASV GP requires both OST isoforms.

**FIG 4:**
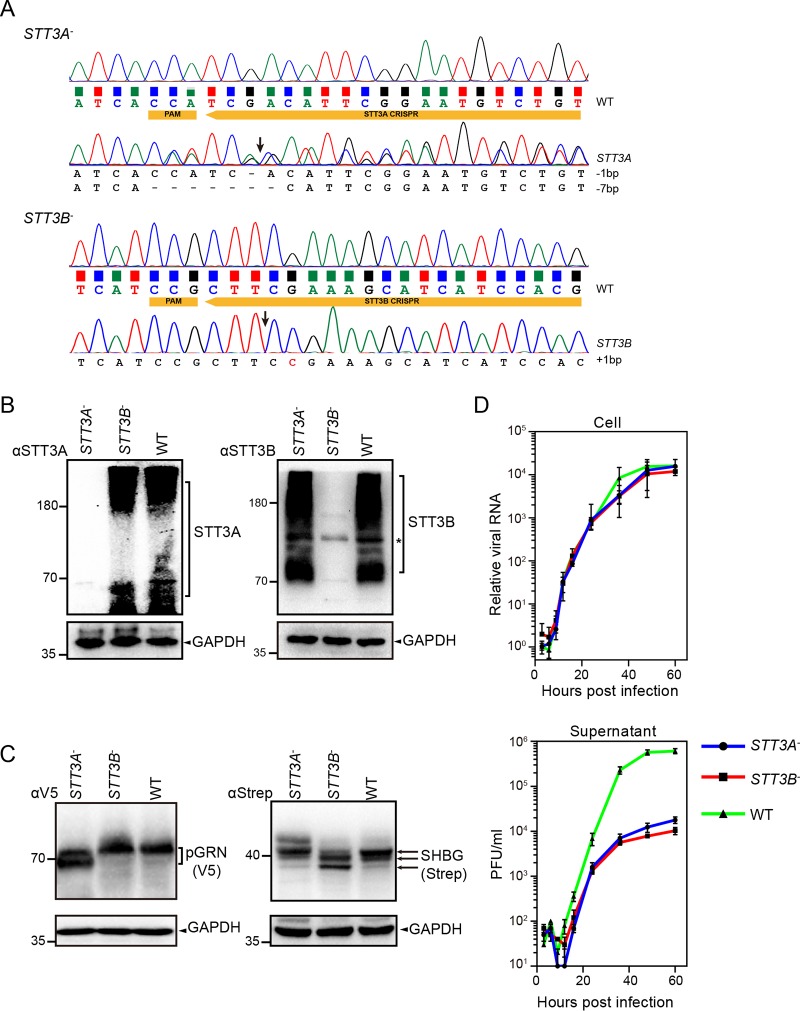
CRISPR-Cas9-mediated isoform-specific knockouts of the OST complex impaired the propagation of the rLCMV/LASV GPC virus. (A) Genotypes of the two selected *STT3A* and *STT3B* knockout cells, as determined by Sanger sequencing of genomic PCR fragments. WT cells were also sequenced as a reference. The PAM sequence and sgRNA sequence targeting gene exons are shown in yellow boxes. Putative cleavage sites of Cas9 are also indicated by arrows. Mutations of knockout cells are listed at the bottom. Only cells displaying frameshift mutations were chosen as candidate knockout cell lines. Although HEK293T cells have three copies of the *STT3A* and *STT3B* genes, the observed number of alleles for *STT3A* and *STT3B* could be less than three. (B) Confirmation of the knockout cell lines by SDS-PAGE and Western blot analysis with antibodies specific to STT3A and STT3B. Lysates of WT cells were also loaded as a reference. (C) The glycosylation patterns of V5-tagged pGRN and Strep-tagged SHBG in *STT3A*^–^, *STT3B*^–^, and WT cells were determined by SDS-PAGE and Western blot analysis with antibodies specific to the V5 and Strep tags, respectively. Different glycoforms are indicated by arrows. GAPDH was also detected as a control for each lane. (D) Growth kinetics of the rLCMV/LASV GPC virus in *STT3A* and *STT3B* knockout cells and WT cells. Cells infected with the rLCMV/LASV GPC virus at an MOI of 0.01 were harvested at the indicated time points. Cellular virus levels were determined by the level of viral S (+) genome RNA normalized to the cellular GAPDH mRNA levels, which were measured by qRT-PCR. The supernatant virus levels were determined by the immunological plaque assay.

Since the small interfering RNA (siRNA)-mediated knockdown of OST subunits in HEK293 cells is reported to be relatively ineffective (53), we generated *STT3A* and *STT3B* knockout cells (*STT3A*^–^ and *STT3B*^–^ cells) by using the CRISPR-Cas9 system to explore the roles of STT3A and STT3B in arenavirus propagation. Plasmids encoding single-guide RNAs (sgRNAs) targeting the exons of the *STT3A* and *STT3B* genes were transfected into HEK293T cells to direct genome editing. Knockouts of *STT3A* and *STT3B* were confirmed by sequencing (Fig. 4A, frameshift mutations at the corresponding sites) and Western blot analysis (Fig. 4B). To further confirm the loss of STT3A and STT3B catalytic activity, the N-glycosylation of two plasmids expressing progranulin (pGRN) or sex hormone-binding globulin (SHBG), which were reported to be specific N-glycosylation substrates of STT3A or STT3B, respectively, was examined (54, 55). As shown in Fig. 4C, N-glycosylation of pGRN was inhibited in *STT3A*^–^ cells but not in wild-type (WT) cells or *STT3B*^–^ cells, whereas knockout of *STT3B* inhibited the N-glycosylation of only SHBG.

To explore whether STT3A and STT3B affect viral replication, we rescued a recombinant arenavirus, rLCMV/LASV GPC, in which LCMV GP was replaced by LASV GP, while the backbone of LCMV was not altered. This recombinant virus can be manipulated in BSL-2 conditions and reportedly exhibits high fitness with intact characteristics of LASV GP (56). *STT3A*^–^ and *STT3B*^–^ cells were infected with rLCMV/LASV GPC and harvested at the indicated time points. The intracellular viral level was determined based on the amount of viral S genomic RNA(+), as determined by quantitative reverse transcription-PCR (qRT-PCR), and the viral titer in the supernatant was determined by an immunological plaque assay. As shown in Fig. 4D, the intracellular level of the viral S genome was only slightly reduced in *STT3A*^–^ or *STT3B*^–^ cells, indicating that viral genome replication was not significantly affected by the knockout of *STT3A* or *STT3B*. The titers in the supernatants of *STT3A*^–^ and *STT3B*^–^ cells were reduced dramatically, indicating that knockout of *STT3A* or *STT3B* impaired the propagation of rLCMV/LASV GPC. These results showed that both STT3A and STT3B are host factors on which the virus depends.

### Knockout of *STT3A* or *STT3B* led to the formation of viral particles with reduced infectivity.

**FIG 5:**
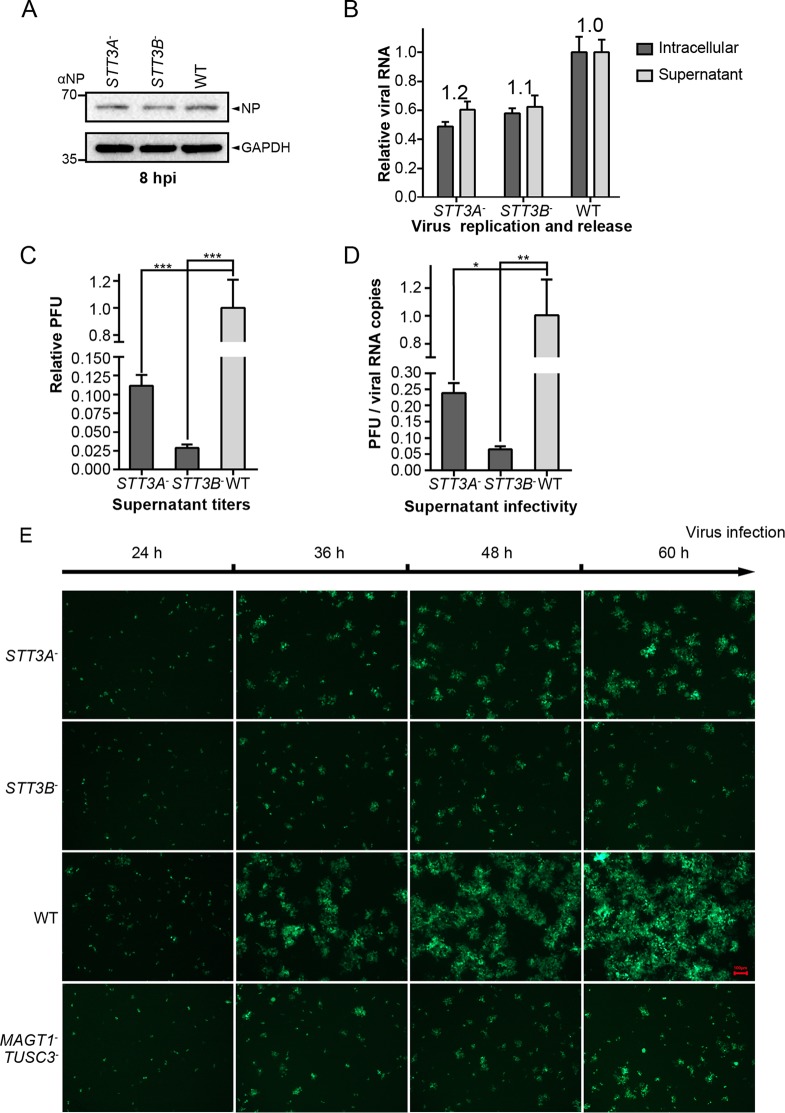
Knockout of *STT3A* or *STT3B* led to the formation of viral particles with reduced infectivity. (A) Single-step infection assay was performed to assess the productive entry and early infection of the rLCMV/LASV GPC virus in *STT3A*^–^ and *STT3B*^–^ cells. The cells were infected with virus at an MOI of 0.01, incubated for 30 min at 4°C to synchronize the entry process, and then shifted to 37°C to allow penetration. Cells were harvested at 8 hpi, and the viral NP level was detected by Western blotting with anti-NP serum. (B) Intracellular and supernatant genome virus levels were measured at 48 hpi to assess virus budding ability by qRT-PCR. The ratio of the supernatant viral genome level versus the intracellular viral genome level was calculated as an indicator of virus budding ability (presented above the column). (C) The relative PFU in the supernatants of different cells at 48 hpi are shown. (D) The ratios of viral PFU versus viral genome RNA copy numbers were calculated as an indicator of viral infectivity. All data displayed here represent the means ± the standard deviations (SD) of three independent experiments, and each independent experiment had two replicates. *, *P* < 0.05; **, *P* < 0.01; ***, *P* < 0.001. (E) Viral foci were observed by fluorescence microscopy at different time points in cells infected with the rLCMV (NP-P2A-GFP)/LASV GPC virus. The foci in *STT3A*^–^ and *STT3B*^–^ cells were notably smaller than those in WT cells. All of the micrographs displayed here were collected at the same scale (the length of the red bar represents 100 μm) and are representatives of several images.

To explore whether STT3A or STT3B plays roles in productive viral entry and early infection, *STT3A*^–^, *STT3B*^–^, and WT cells were infected with rLCMV/LASV GPC at a multiplicity of infection (MOI) of 0.01. We examined intracellular levels of viral nucleoprotein (NP) at 8 h postinfection (hpi), at which time point LASV has reportedly finished entry (57). As shown in Fig. 5A, no significant changes were observed, suggesting that knockout of STT3A or STT3B did not affect viral entry or early infection.

Since knockout of STT3A or STT3B had no significant effects on the replication of the rLCMV/LASV GPC genome (Fig. 4D), we tested whether knockout of *STT3A* or *STT3B* affected the release of viral particles. To this end, *STT3A*^–^ or *STT3B*^–^ cells were infected with rLCMV/LASV GPC, and both intracellular and supernatant viral genomes were measured by qRT-PCR at 48 hpi. The ratio of the supernatant viral genome level versus the intracellular viral genome level was calculated as an indicator of virus budding ability. Knockout of STT3A or STT3B had no significant effect on virus budding ability (Fig. 5B), suggesting that *STT3A* or *STT3B* did not affect viral release. Although the absolute viral genome levels were partly reduced in *STT3A*^–^ and *STT3B*^–^ cells, the extent of reduction (<2-fold) was insufficient to explain the dramatic reduction in viral titers in the supernatants (>30-fold reduction, shown in Fig. 4D).

The viral titers decreased dramatically with only mild reductions in the viral genome RNA levels in the supernatants of *STT3A*^–^ or *STT3B*^–^ cells (Fig. 5C), suggesting that viral particles derived from *STT3A*^–^ or *STT3B*^–^ cells may have reduced viral infectivity. We calculated the ratio of viral titers to viral genome RNA copy numbers in the supernatant as an indicator of viral infectivity. As shown in Fig. 5D, viral particles derived from *STT3A*^–^ or *STT3B*^–^ knockout cells exhibited reduced viral infectivity. Viral infectivity was further visualized by the infection of a rLCMV (NP-P2A-GFP)/LASV GPC virus, in which LCMV NP was linked with GFP by a P2A self-cleaving peptide. Cells infected with the rLCMV (NP-P2A-GFP)/LASV GPC virus were immobilized, and viral foci were observed at different time points. As shown in Fig. 5E, knockout of *STT3A* and *STT3B* did not reduce the number of viral foci; however, the viral foci in *STT3A*^–^ and *STT3B*^–^ cells were notably smaller than those in WT cells, suggesting reduced virus infectivity. Based on these results, we concluded that the inhibited propagation of the rLCMV/LASV GPC virus by the knockout of *STT3A* or *STT3B* was mainly due to the reduced viral infectivity.

### Knockout of *STT3B* caused hypoglycosylation of LASV GP.

**FIG 6:**
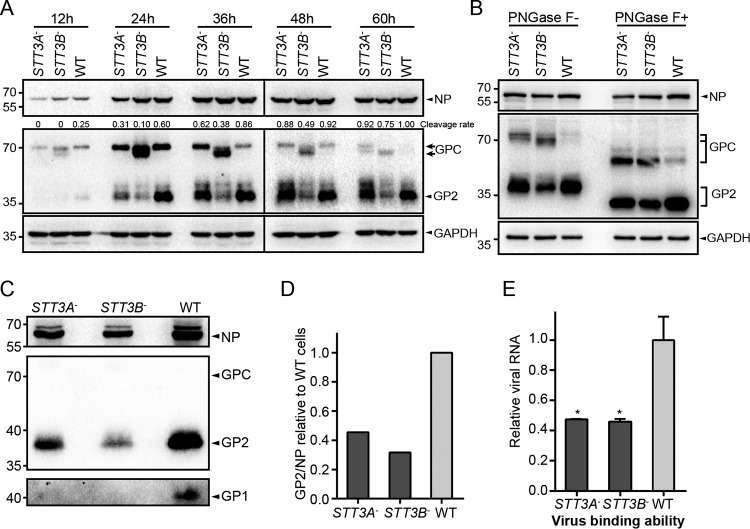
Knockout of *STT3B* caused hypoglycosylation of LASV GP and reduced its expression level. (A) Expression and glycosylation profiles of LASV GP in *STT3A*^–^, *STT3B*^–^ cells and WT cells. Cells infected with the rLCMV/LASV GPC virus at an MOI of 0.01 were harvested at the indicated time points and subjected to Western blot analysis with GP2-specific serum. Viral NP was also detected by a specific antiserum, serving as an internal control to reflect the replication level of the virus. The cleavage rates of tagged and untagged GP2 are shown above as determined by densitometric quantification. (B) The reduced molecular weight of viral GP in *STT3B*^–^ cells was due to a deficiency in glycans. Viral proteins from different cell lines were subjected to cleavage by PNGase F and Western blot analysis with GP2-specific serum. Viral NP was also detected. (C) Quantitation of viral glycoprotein incorporated into viral particles. Viral particles (supernatant) derived from different cell lines at 48 hpi were collected by ultracentrifugation and screened for viral proteins (GP1, GP2, and NP) by Western blotting. (D) The levels of incorporated proteins were determined by the band intensities, and the ratios of viral GP2 versus viral NP were calculated as an indicator of the level of viral glycoproteins incorporated into a single viral particle. SDs are not shown because viral particles were derived from one individual preparation. In panels A to C, GAPDH was also detected as a loading control for each lane. (E) The cell binding ability of RNA-normalized viruses derived from different cell lines to normal BHK-21 cells was measured by qRT-PCR and normalized to the GAPDH mRNA level. The data displayed here represent the means ± the SD of three independent experiments, and each independent experiment had two replicates.

Since both STT3A and STT3B play roles in virus propagation, we sought to investigate the glycosylation profile of viral GP in *STT3A*^–^ and *STT3B*^–^ cells. Cells infected with the rLCMV/LASV GPC virus were harvested at the indicated time points and subjected to Western blotting. In *STT3A*^–^ cells, the viral GP displayed the same glycosylation pattern as that in WT cells, but the proteolytic cleavage process was hindered in the early stage of infection (24 and 36 hpi). Even so, the viral GP in *STT3A*^–^ cells reached a level of maturation similar to that observed in WT cells at the late stage of infection (Fig. 6A). Knockout of *STT3A* did not significantly affect the glycosylation of LASV GP, which might be explained by the functionally compensatory effect executed by the STT3B isoform of OST in *STT3A*^–^ cells. However, we observed an obviously hypoglycosylated form of noncleaved GPC in *STT3B*^–^ cells. To exclude the possibility that the reduced molecular weight of viral GPC was caused by other modifications, such as phosphorylation, viral proteins from different cell lines were subjected to cleavage by PNGase F to remove the N-glycans. As shown in Fig. 6B, the migration of the unglycosylated form of noncleaved GPC was identical among these cells, suggesting that the differential migration of the glycosylated form of GPC in *STT3B*^–^ cells was due to the distinct glycosylation status of GPC. Taken together, these data showed that LASV GP was a preferential substrate of STT3B-OST and that the full glycosylation of LASV GP requires the STT3B-OST isoform. Although STT3A was not an essential prerequisite for the glycosylation of LASV GP, loss of STT3A delayed its proteolytic cleavage.

### Incorporation of viral GP into viral particles was reduced in *STT3A*^–^ and *STT3B*^–^ cells.

Since knockout of *STT3A* or *STT3B* hindered the proteolytic cleavage of GPC, we explored whether the incorporation of GP in virions was affected in *STT3A*^–^ and *STT3B*^–^ cells. Virions derived from different cells were collected and screened for viral proteins (GP1, GP2, and NP) by Western blotting. As shown in Fig. 6C, only cleaved GP1 or GP2 was detected, which was in line with a previous report demonstrating that cleavage is necessary for the incorporation of GP into virions (14). Viral NP, GP1, and GP2 levels were reduced in viral particles derived from *STT3A*^–^ and *STT3B*^–^ cells, suggesting reduced virus production. The GP2/NP band intensity ratios were calculated as an indicator of the number of viral GPs incorporated into a single viral particle. As shown in Fig. 6D, the ratios of GP2 to NP derived from both *STT3A*^–^ and *STT3B*^–^ cells were significantly reduced compared to those from WT cells, suggesting that the GP composition in virions was reduced.

To explore whether the reduced GP levels in virions derived from *STT3A*^–^ or *STT3B*^–^ cells affect virus infection, the binding abilities of these virions were tested. Genome RNA-normalized virions derived from different cells were added to BHK-21 cells, and the virus binding ability was measured by qRT-PCR. As shown in Fig. 6E, virions derived from *STT3A*^–^ and *STT3B*^–^ cells exhibited a partial defect in their ability to attach to normal BHK-21 cells. Based on these results, we concluded that knockout of *STT3A* and *STT3B* reduced the level of GP incorporated into viral particles and hindered the ability of the virus to bind permissive cells.

### MAGT1 and TUSC3 were required for the full glycosylation of LASV GP.

The results [resented above revealed that LASV GP was a preferred substrate of STT3B-OST. MAGT1 and TUSC3 are specific subunits of the STT3B-OST complex. Despite their replaceable functions, MAGT1 mRNA was shown to be widely expressed in human tissues, whereas the expression of TUSC3 is relatively more restricted (58). The expression of TUSC3 in several cultured cell lines was reported to be difficult to detect by Western blotting (53). MAGT1 was found to weakly interact with LASV GP in our MS data set, while TUSC3 was not identified (Table S1). This result could be explained by previous reports demonstrating that MAGT1 and TUSC3 easily dissociated from the canine OST during the purification process (52). To confirm that MAGT1 and TUSC3 were host interactors of LASV GP, a pCAGGs plasmid encoding 6×His-tagged MAGT1 or Flag-tagged TUSC3 was cotransfected with LASV GP into HEK293T cells. Coimmunoprecipitation and Western blot analyses revealed that viral GP but not GFP was able to pull down exogenously expressed MAGT1 and TUSC3, indicating that these proteins were also host interactors of LASV GP (Fig. 3G and H).

**FIG 7:**
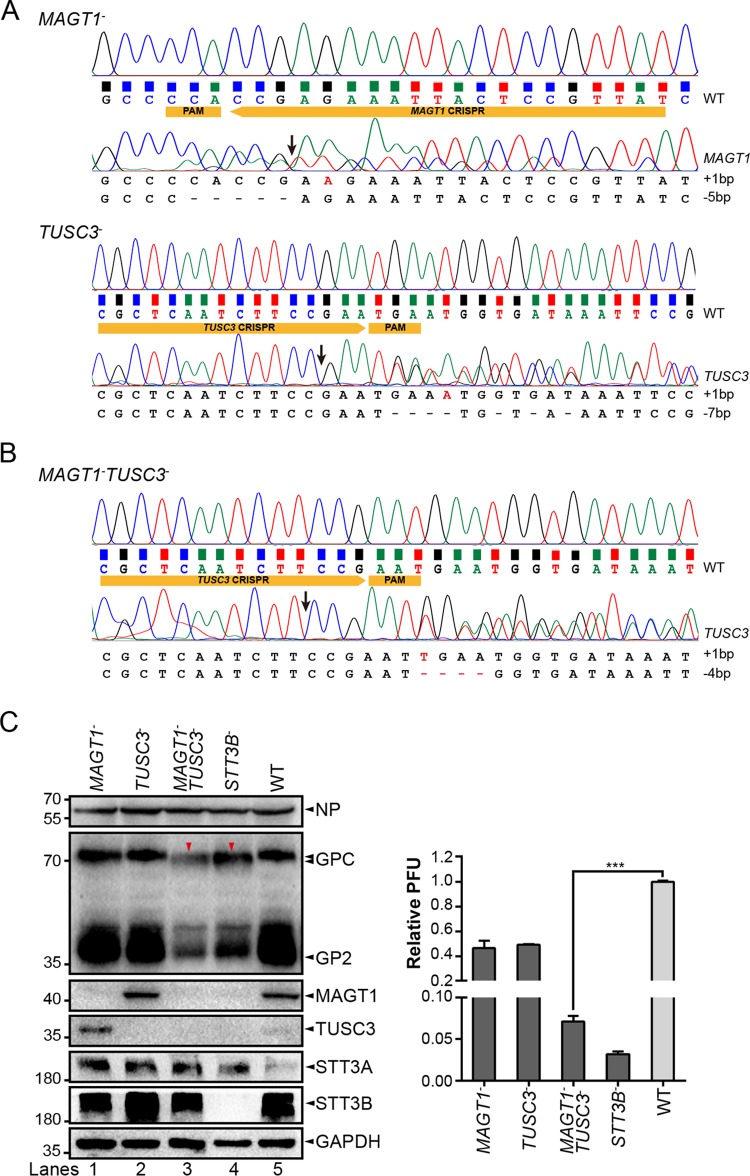
MAGT1 and TUSC3 were required for the full glycosylation of LASV GP. Genotypes of *MAGT1*^–^ and *TUSC3*^–^ cells (A) and *MAGT1*^–^ *TUSC3*^–^ cells (B), as determined by Sanger sequencing of genomic PCR products. WT cells were also sequenced as a reference. The PAM sequence and sgRNA targeting gene exons are shown in yellow boxes. Putative cleavage sites of Cas9 are also indicated by arrows. Mutations of knockout cells are listed at the bottom. Only cells displaying frameshift mutations were chosen as candidate knockout cell lines. Although HEK293T cells have seven copies of the *MAGT1* gene and two copies of the *TUSC3* gene, the observed number of alleles for *MAGT1* and *TUSC3* could be less than their copy numbers. (C) Expression and glycosylation profiles of LASV GP in infected *MAGT1*^–^, *TUSC3*^–^, and *MAGT1*^–^ *TUSC3*^–^ cells or WT cells as a control. Cells infected with the rLCMV/LASV GPC virus at an MOI of 0.01 were harvested at 36 hpi and subjected to Western blot analysis with GP2-specific serum. Knockouts of *MAGT1* and *TUSC3* were confirmed by their specific antibodies. NP, STT3A, and STT3B were also detected. GAPDH was detected as a loading control for each lane. Hypoglycosylation of viral glycoprotein is indicated by red triangles. Supernatant viral titers derived from different cell lines were determined by the immunological plaque assay. The data presented here represent the means ± the SD of three independent experiments, and each independent experiment had two replicates.

We then sought to explore the roles of MAGT1 and TUSC3 in virus propagation. CRISPR-Cas9-mediated knockout of *MAGT1* and *TUSC3* cell lines were constructed (*MAGT1*^–^ and *TUSC3*^–^ cells) as described above and confirmed by sequencing (Fig. 7A) and Western blot analysis (Fig. 7C). The expression levels of both MAGT1 and TUSC3 were also decreased in *STT3B*^–^ cells, indicating that their expression relies on the existence of STT3B. Although virus titers were partly reduced in *MAGT1*^–^ and *TUSC3*^–^ cells compared to that in WT cells (Fig. 7C, right), glycosylation of LASV GP was not significantly altered in these two cells. The inefficiency of functional knockout might be explained by the functional compensation between the two proteins. The level of TUSC3 in WT cells was almost undetectable by Western blotting, while a deficiency of MAGT1 was accompanied by obviously increased expression of TUSC3 (Fig. 7C, lanes 1 and 5). To confirm this finding, the *MAGT1*^–^*TUSC3*^–^ cell line was constructed (Fig. 7B). Both *MAGT1*^–^ *TUSC3*^–^ cells and *STT3B*^–^ cells were then infected with the rLCMV/LASV GPC virus, and we observed the same hypoglycosylation pattern of viral GP in the two cells compared to the glycosylation pattern in WT cells (Fig. 7C), indicating that MAGT1 or TUSC3 was required for full viral GP glycosylation. The virus titer derived from *MAGT1*^–^*TUSC3*^–^ cells was also dramatically reduced compared to that derived from WT cells (Fig. 7C). Similarly, the viral foci in *MAGT1*^–^ *TUSC3*^–^ cells were notably smaller than those in WT cells (Fig. 5D), as in *STT3B*^–^ cells.

### Full glycosylation of LASV GP requires the CXXC active-site motifs of MAGT1 and TUSC3.

**FIG 8:**
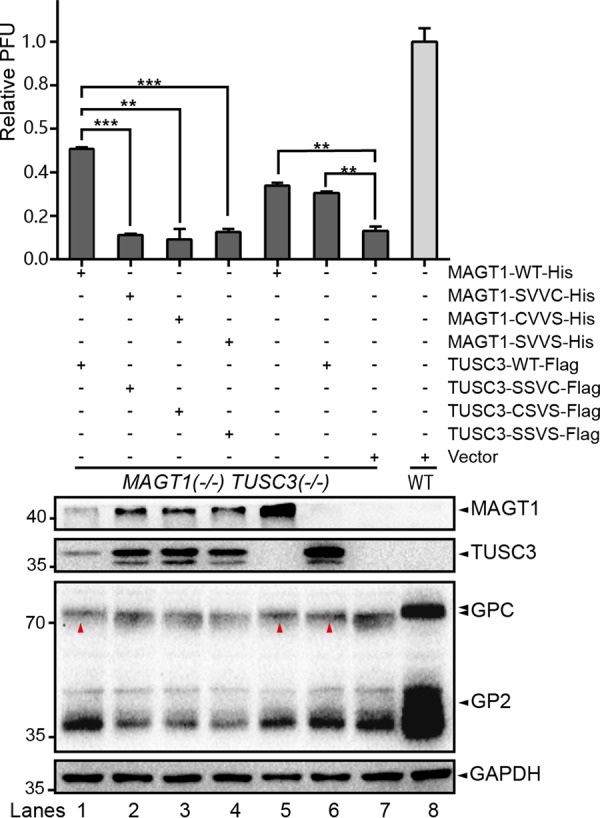
Full glycosylation of LASV GP requires the CXXC active site motifs of MAGT1 and TUSC3. Plasmids encoding MAGT1 and TUSC3, or MAGT1 and TUSC3 with SXXC or CXXS or SXXS mutations, were transfected into *MAGT1*^–^ *TUSC3*^–^ cells to restore the glycosylation of LASV GP. At 24 h posttransfection, the cells were infected with the rLCMV/LASV GPC virus at an MOI of 0.01. Cells and supernatants were harvested at 36 hpi. The glycosylation patterns of viral GP were analyzed by Western blotting with serum specific to viral GP2. The expression of MAGT1 and TUSC3 was confirmed by specific antibodies. GAPDH was detected as a loading control for each lane. Restoration of virus glycosylation is indicated by red triangles. Supernatant viral titers derived from different cell lines were determined by the immunological plaque assay. The data presented here represent the means ± the SD of three independent experiments, and each independent experiment had two replicates.

MAGT1 and TUSC3 are thioredoxin homologs, and a luminally oriented CXXC active-site motif is responsible for their oxidoreductase activity (59). A protein tertiary structure stabilized by disulfide bonds is a negative regulatory factor for their posttranslational glycosylation (60). MAGT1 and TUSC3 delay the folding of nascent polypeptides by forming mixed disulfide bonds with cysteine-proximal glycosylation sequons via their CXXC site motifs and thereby facilitate access to STT3B (41). Mutation of the active site cysteines of MAGT1 or TUSC3 resulted in the hypoglycosylation of a subset of STT3B-dependent substrates (41). To test whether the glycosylation of LASV GP requires the oxidoreductase activity of MAGT1 and TUSC3, plasmids encoding MAGT1/TUSC3 or MAGT1/TUSC3 with SXXC, CXXS, or SXXS mutations were transfected into *MAGT1*^–^ *TUSC3*^–^ cells to restore the glycosylation of LASV GP. As shown in Fig. 8, the expression of WT MAGT1 or WT TUSC3 was able to restore the glycosylation of LASV GPC (lanes 1, 5, and 6), whereas MAGT1 and TUSC3 with mutations of any cysteine in their CXXC site motifs failed to restore glycosylation and virus replication (lanes 2, 3, and 4). Our results revealed that the oxidoreductase CXXC active-site motifs of MAGT1 and TUSC3 play roles in the full glycosylation of LASV GP.

### A small-molecule OST inhibitor impaired the propagation of the rLCMV/LASV GPC virus.

**FIG 9:**
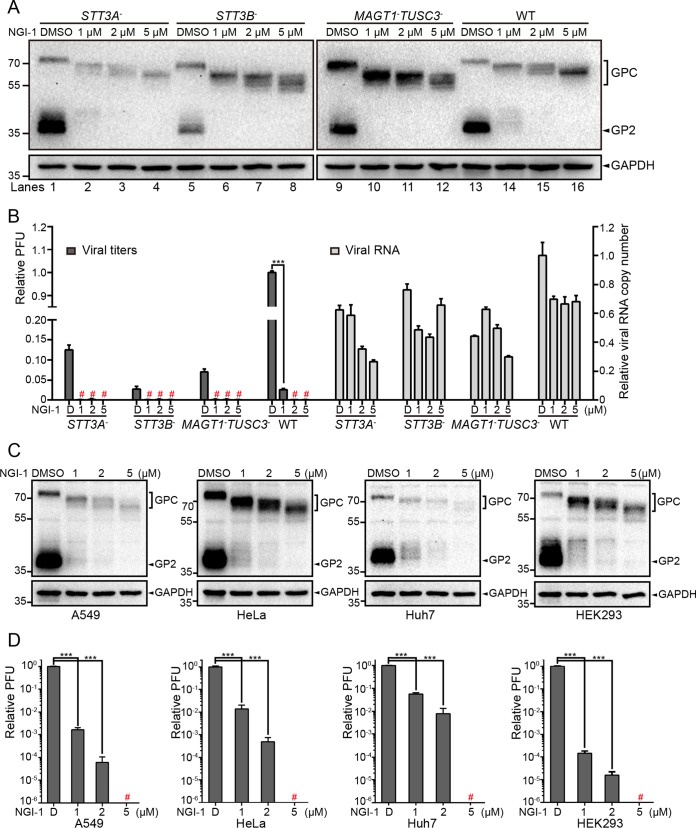
A small-molecule OST inhibitor impaired the propagation of the rLCMV/LASV GPC virus. (A) *STT3A*^–^, *STT3B*^–^, *MAGT1*^–^ *TUSC3*^–^, and WT (HEK293T) cells were infected with the rLCMV/LASV GPC virus at an MOI of 0.01. NGI-1 at the indicated concentrations was added to cells after virus entry. Dimethyl sulfoxide (DMSO) was added as a control. Cells and supernatants were harvested at 36 hpi. The glycosylation patterns of viral glycoprotein were determined by Western blotting with GP2-specific serum. GAPDH was also detected as a loading control for each lane. (B) Supernatant viral titers and viral RNA copy numbers were determined by the immunological plaque assay and qRT-PCR, respectively. Viral titers less than 10 PFU/ml were considered undetected and are marked with a red number sign (“#”). (C) A549, HeLa, Huh7, and HEK293 cells were infected with the rLCMV/LASV GPC virus at an MOI of 0.01. NGI-1 at the indicated concentrations were added to cells after virus entry. DMSO was added as a control. Cells and supernatants were harvested at 36 hpi. The glycosylation patterns of viral glycoprotein were determined by Western blotting with GP2-specific serum. GAPDH was also detected as a loading control for each lane. (D) Supernatant viral titers were determined by the immunological plaque assay and qRT-PCR, respectively. Viral titers of <10 PFU/ml were considered undetected and are marked with a red number sign (“#”).

NGI-1, a small-molecule OST inhibitor, was reported to directly target and block the function of the OST catalytic subunits STT3A and STT3B. Unlike tunicamycin, NGI-1 does not completely inhibit all the N-glycosylation sites, and this probably determines the reduced cellular toxicity of NGI-1, making it an excellent approach to modulate N-glycosylation in mammalian cells (61). To test the inhibitory effect of NGI-1 on the N-glycosylation of LASV GP, *STT3A*^–^, *STT3B*^–^, *MAGT1*^–^ *TUSC3*^–^, and WT cells were infected with the rLCMV/LASV GPC virus and then treated with NGI-1 at the indicated concentrations. None of the concentrations showed cellular toxicity during the incubation time (data not shown). Cells were harvested at 36 hpi and subjected to Western blot analysis. As shown in Fig. 9A, NGI-1 was able to inhibit the glycosylation of LASV GP mediated by STT3A-OST (in *STT3B*^–^ and *MAGT1*^–^ *TUSC3*^–^ cells) or STT3B-OST (in *STT3A*^–^ cells) and impaired its proteolytic cleavage in a dose-dependent manner. At the same NGI-1 concentrations, hypoglycosylation of LASV GP was most severe in *STT3B*^–^ and *MAGT1*^–^ *TUSC3*^–^ cells compared to that in *STT3A*^–^ and WT cells.

To further evaluate the effect of NGI-1 on virus propagation, supernatant viral titers and viral RNA copy numbers were determined by the immunological plaque assay and qRT-PCR, respectively. As shown in Fig. 9B, NGI-1 treatment significantly reduced the viral titers, while the viral RNA copy numbers were only mildly reduced, indicating that NGI-1 mainly impaired virus infectivity but not the number of released progeny viruses. The effect of NGI-1 on LASV GP and virus propagation was also tested in several other human cell lines, including A549, HeLa, Huh7, and HEK293 cells, and both hypoglycosylation of LASV GP and reduced viral titer were observed (Fig. 9C and D), which is consistent with the findings in HEK293T cells.

### The preferential requirement for STT3B-dependent N-glycosylation of GP was conserved among arenaviruses.

**FIG 10:**
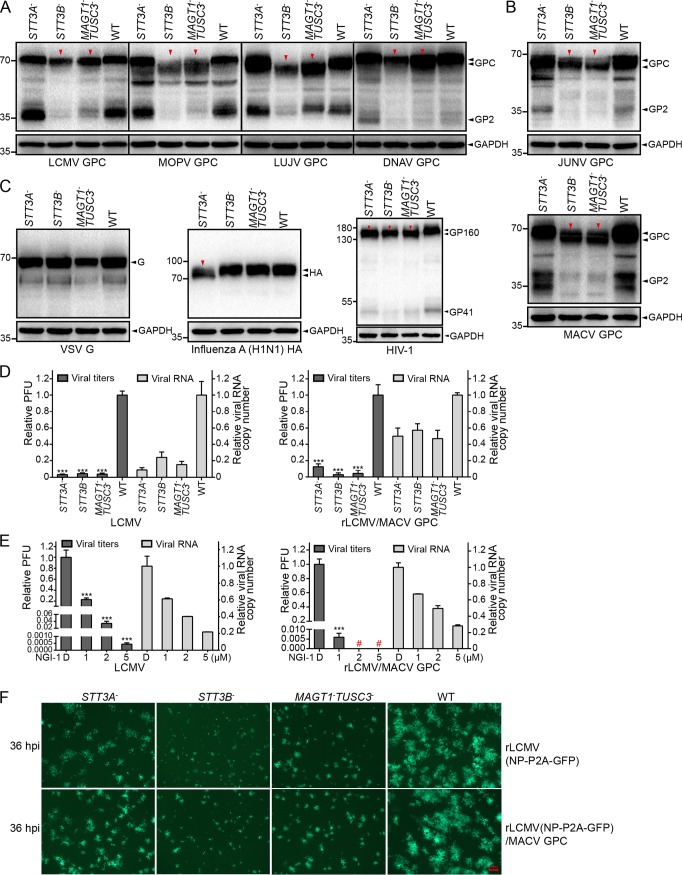
The preferential requirement for STT3B-dependent N-glycosylation was conserved among arenavirus GPs. pCAGGs plasmids encoding Strep-tagged glycoproteins of four OW arenaviruses (A) and two NW arenaviruses (B) or VSV-G, influenza A virus HA, and HIV-1 GP160 (C) were transfected into *STT3A*^–^, *STT3B*^–^, *MAGT1*^–^ *TUSC3*^–^, and WT cells. Cells were harvested at 24 h posttransfection and subjected to Western blot analysis with antibodies specific to the Strep epitope. Hypoglycosylation patterns of virus glycoprotein are indicated by red triangles. GAPDH was also detected as a loading control for each lane. (D) The supernatant viral titers and viral RNA copy numbers of LCMV and rLCMV/MACV GPC viruses in *STT3A*^–^, *STT3B*^–^, *MAGT1*^–^ *TUSC3*^–^, and WT cells were determined by the immunological plaque assay and qRT-PCR, respectively. (E) The supernatant virus titers and viral RNA copy numbers of the LCMV and rLCMV/MACV GPC viruses in HEK293T cells treated with the indicated concentrations of NGI-1. DMSO was added as a control. Viral titers of <10 PFU/ml were considered undetected and are marked with a red number sign (“#”). (F) Viral foci were observed by fluorescence microscopy at 36 hpi in cells infected with the rLCMV (NP-P2A-GFP) and rLCMV (NP-P2A-GFP)/MACV GPC viruses. The viral foci in OST-knockout cells, especially in *STT3B*^–^ and *MAGT1*^–^ *TUSC3*^–^ cells, were notably smaller than those in WT cells. All the micrographs displayed here were collected at the same scale (the length of the red bar represents 100 μm) and are representatives of several images.

Arenavirus GPs are all highly glycosylated. We next sought to explore whether the preferential requirement for STT3B-dependent N-glycosylation is conserved among other arenaviruses. Plasmids encoding GPs of OW arenaviruses, including LCMV, Mopeia virus (MOPV), Lujo virus (LUJV), and Dandenong virus (DNAV) (Fig. 10A) and NW arenaviruses, including JUNV and MACV (Fig. 10B), were transfected into *STT3A*^–^, *STT3B*^–^, *MAGT1*^–^ *TUSC3*^–^, and WT cells. The glycosylation patterns of viral GPs were then analyzed by Western blotting. We observed hypoglycosylation patterns for all the arenavirus GPs tested in *STT3B*^–^ and *MAGT1*^–^ *TUSC3*^–^ cells, indicating the conserved requirement of STT3B and MAGT1/TUSC3 for arenavirus glycosylation.

We also tested the effect of knockout of *STT3A* or *STT3B* on the glycoproteins of vesicular stomatitis virus (VSV), influenza A virus, and human immunodeficiency virus 1 (HIV-1). As shown in Fig. 10C, influenza HA was only affected by knockout of *STT3B*, whereas HIV-1 envelope glycoprotein (GP160) was affected by knockout of *STT3A* or *STT3B*. No significant changes of molecular weight were observed in VSV glycoprotein (VSV-G).

To test whether STT3A and STT3B can affect the infectivity of other arenaviruses, two other viruses, LCMV and rLCMV/MACV GPC, were chosen as representatives of OW and NW arenaviruses to explore their propagation in *STT3A*^–^, *STT3B*^–^, *MAGT1*^–^ *TUSC3*^–^, and WT cells. Cells were infected with viruses and harvested at 36 hpi. Supernatant viral titers and viral RNA copy numbers were determined by the immunological plaque assay and qRT-PCR, respectively. As shown in Fig. 10D, both the titers and the RNA copy numbers of the LCMV and rLCMV/MACV GPC viruses were reduced in the three OST-knockout cells, demonstrating the impaired propagation of both viruses. Even so, the viral RNA copy numbers were reduced less than the viral titers, especially for the rLCMV/MACV GPC virus, indicating that virus infectivity was affected, which is in line with that of the rLCMV/LASV GPC virus. NGI-1 was also able to severely reduce the viral titers, whereas viral RNA copy numbers were only mildly reduced (Fig. 10E). The viral infectivity was further visualized by the infection of the rLCMV (NP-P2A-GFP) and rLCMV (NP-P2A-GFP)/MACV GPC viruses. Similarly, the viral foci (36 hpi) in OST-knockout cells were notably smaller than those in WT cells, especially in *STT3B*^–^ and *MAGT1*^–^ *TUSC3*^–^ cells (Fig. 10F). Our results revealed that the dependence on the host OST complex was conserved among arenaviruses.

## DISCUSSION

Arenavirus GPs play critical roles in the viral life cycles. They facilitate viral entry via binding to the virus receptor at the extracellular membrane or promoting membrane fusion in the endosome and participate in virus budding via interacting with the virus matrix protein Z to be incorporated into the envelope of nascent virions. Arenavirus GP is first translated into the ER lumen as a GPC precursor, which is mediated by SSP, and then undergoes a series of folding and modification processes along the secretory pathway to achieve a mature and functional conformation. Among these processes, N-glycosylation in the ER lumen is the most dominant modification of LASV GP. We systematically identified the host interactors of LASV GP using the AP-MS approach. This methodology enables us to gain detailed insights into the host machinery involved in stages of LASV GP synthesis, processing, and transport through the secretory pathway. Proteomic analysis identified multiple proteins of the OST complex as interactors of LASV GP. This was consistent with a previous interactome of LCMV GP (Table S1), although their roles were not examined (49). The congruence between the interactomes of LASV and LCMV GPs highlights the reliability of these observations and prompted us to investigate the conserved roles of the OST complex in arenaviruses.

Although most viral membrane proteins are glycoproteins, the relationship between viruses and the OST complex has not been investigated in detail, except for flavivirus. A global host interactome analysis and a pooled CRISPR genetic screening revealed that the OST complex interacts with dengue virus (DENV) nonstructural proteins (NS1, NS3, and NS4B) (62) and is crucially involved in DENV RNA replication independent of its canonical roles in N-linked glycosylation (63). The oxidoreductase activity of STT3B-OST was also reported to be necessary for its propagation (64). Our further functional study indicated that the OST complex is a host factor on which the arenaviruses depend, suggesting that the OST complex might broadly participate in host-virus interactions.

We found that knockout of *STT3B* caused hypoglycosylation of viral GPC, while knockout of *STT3A* did not have such an effect (Fig. 6A), suggesting a preferential requirement for the STT3B isoform in the glycosylation of LASV GP. This result shows characteristics that differ from those of flavivirus, since DENV NS1 and NS4B were shown to be fully glycosylated in STT3A or STT3B knockout cells (63, 64), and only simultaneous knockdown of STT3A and STT3B causes a major glycosylation defect of NS1 (62). Different dependencies of STT3A and STT3B for N-glycosylation were also found in VSV-G, influenza HA, and HIV-1 GP160 proteins, indicating the diverse mechanisms of modification utilized by different viruses (Fig. 10C). Nevertheless, both STT3A and STT3B were shown to be directly or indirectly required for the formation of infectious rLCMV/LASV GPC viruses but did not significantly influence the number of progeny viruses released (Fig. 5C and D). Virions released from *STT3A*^–^ and *STT3B*^–^ cells showed reduced incorporation of GPs into virion particles and a partly reduced binding ability to normal permissive cells (Fig. 6E). NGI-1, a small-molecule OST inhibitor, was able to strongly inhibit virus propagation (Fig. 9A) by affecting virus infectivity (Fig. 9B) without resulting in significant cytotoxicity. Thus, pharmacological inhibition of the host OST complex by NGI-1 may provide a useful treatment option to efficiently inhibit virus spread and reduce the fatality rate caused by Lassa fever in the future.

The relative importance of the STT3B isoform of OST in LASV infection may be related to the distinct enzymatic mechanisms of the two isoforms. In mammalian cells, the OST complex is a hetero-oligomeric complex consisting of a catalytically active subunit with several noncatalytic auxiliary subunits (39). The additional components either play a structural role to support the structural stability and enzymatic activity of the OST complex or modulate the N-glycosylation efficiency for certain precursors (65, 66). *STT3A* and *STT3B* arose from the same ancestral *STT3* gene during the evolution of multicellular eukaryotes and share a 59% amino acid sequence identity (39). Both STT3A and STT3B are widely expressed in human tissues, and the full glycosylation of polypeptides involves the cooperation of the two isoforms. The STT3A-OST isoform is primarily responsible for the cotranslational modification of sequons as nascent polypeptide chains enter the lumen of the rough endoplasmic reticulum (RER) through direct interaction with the Sec61 translocon complex (51, 67, 68). Most acceptor sites in host glycoproteins are modified by the cotranslational pathway, and depletion of STT3A by siRNA upregulates the level of the luminal ER chaperone BiP and induces the unfolded protein response in response to the hypoglycosylation of host proteins. Unlike STT3A-OST, the STT3B-OST isoform is less competent for the cotranslational modification and efficiently mediates posttranslational modification of glycosylation sites that are skipped by STT3A after nascent polypeptides enter the ER lumen (51). Here, no significant changes in GP molecular weight were observed in *STT3A*^–^ cells, implying that the glycosylation acceptor sites for STT3A may also be targeted by STT3B, whereas in *STT3B*^–^ cells, the glycosylation acceptor site/sites for STT3B may not be fully rescued by STT3A; thus, the reduction in GP molecular weight was observed, and the GP maturation process and virion infectivity were impaired significantly. These results suggested the existence of glycosylation sites in viral GPC that are prone to being skipped by STT3A-OST and designated to be posttranslationally modified by STT3B-OST. MAGT1 and TUSC3 are specific subunits of STT3B-OST that have overlapping functions. CRISPR-Cas9-mediated knockout of either *MAGTI* or *TUSC3* did not reduce the glycosylation of LASV GP, whereas knockout of both led to the same hypoglycosylation pattern as that observed in *STT3B*^–^ cells, indicating that MAGT1 and TUSC3 exhibit compensatory functions for the glycosylation of LASV GP (Fig. 7B). Site-directed mutagenesis further revealed that the oxidoreductase CXXC active site motif of MAGT1 or TUSC3 was essential for the glycosylation of LASV GP (Fig. 8). This finding provided novel details regarding the virus glycosylation process in the ER lumen.

STT3A and STT3B show relatively differential expression profiles in human tissues; STT3A expression was reported to be very low in the brain, lung, and kidney and high in the placenta, liver, skeletal muscle, and pancreas, whereas STT3B showed more uniform levels of expression (52). Although STT3A and STT3B show tissue-specific expression, this phenomenon likely does not link the preferential requirement for the STT3B-OST isoform with the tissue tropism of virus infection *in vivo*, since STT3A was also here demonstrated to be a host prerequisite for virus propagation by indirectly influencing the infectivity of progeny viruses.

The OST complex utilizes dolichol-linked oligosaccharides (OS-PP-Dol) as donor substrates for the N-linked glycosylation of asparagine residues in the N-X-T/S consensus sites of newly synthesized proteins (69). The fully assembled OS-PP-Dol is Glc_3_Man_9_GlcNAc_2_-PP-Dol, which is assembled on the cytoplasmic face of the RER by glycosyltransferases that mediate the transfer of single glycosyl residues onto the dolichol phosphate step by step (70). Assembly intermediates of OS-PP-Dol with different lengths (Glc_0–2_Man_0–9_GlcNAc_2_-PP-Dol) can also serve as the donor substrate for the OST complex *in vivo* and *in vitro* (71, 72). It was reported that STT3A and STT3B assembled into different OST complexes with a distinct discrimination ability to select Glc_3_Man_9_GlcNAc_2_-PP-Dol over its assembly intermediate Man_9_GlcNAc_2_-PP-Dol, which lacks the terminal glucose residue as the oligosaccharide donor substrate for N-glycosylation (52). The STT3B-OST complex is substantially more active and shows a reduced ability to discriminate different oligosaccharide structures; in contract, the STT3A-OST complex is extremely selective and preferentially glycosylates nascent proteins with the fully assembled Glc_3_Man_9_GlcNAc_2_-PP-Dol (52). It is interesting to associate this distinct substrate-selective characteristic of STT3A and STT3B with the recently reported glycan heterogeneity of LASV GP. Analysis of the LASV glycome composition by hydrophilic interaction chromatography ultraperformance liquid chromatography revealed that the virus possesses an abundance of underprocessed oligomannose-type glycans, which form punctuated clusters to shield the proteinous surface from the humoral immune response (73). It remains unknown whether the site-specific heterogeneity of virion surface glycans has any relevance to the preferential utilization of host OST isoforms by viruses during the glycosylation process. Further studies are needed to deeply elucidate the physiological significance of this host glycosylation strategy utilized by arenaviruses.

## MATERIALS AND METHODS

### Cells.

HEK293T, HEK293, HeLa, A549, Huh7, and BHK-21 cells were obtained from the American Type Culture Collection (ATCC) and maintained in Dulbecco modified Eagle medium (DMEM; Gibco) supplemented with 10% (vol/vol) fetal bovine serum (FBS; Gibco) in a 37°C incubator with 5% CO_2_.

### Plasmids.

The cDNA sequences of GPs of LASV (Josiah strain, GenBank accession no. HQ688672.1), LCMV (Armstrong strain, GenBank AY847350.1), MOPV (AN20410 strain, GenBank JX985097.1), LUJV (GenBank FJ952384.1), DNAV (isolate 0710-2678, GenBank EU136038.1), JUNV (XJ13 strain, GenBank AY358023.2), MACV (Carvallo strain, GenBank KM198592.1), and VSV (GenBank DQ408670.1) were obtained from NCBI and chemically synthesized by Sangon Biotech (China). The cDNA sequences of influenza A virus HA (A/Puerto Rico/8/1934(H1N1), GenBank MH785011.1) and HIV-1 GP160 (isolate NL43clone, GenBank AY669735.1) were gifts from Chen JianJun and Peng Ke, respectively, in our institute. The cDNA sequences of viral GPs and GFP were cloned into pCAGGs plasmids with a C-terminal Twin-Strep-tag (WSHPQFEKGGGSGGGSGGSAWSHPQFEK). pLX304 plasmids encoding V5-tagged RPN1, RPN2, OSTC, DDOST, and pGRN were obtained from a purchased public genome-scale lentiviral expression library of human ORFs (74). pcDNA3.1(–) plasmids encoding 6×His-tagged MAGT1, Flag-tagged TUSC3, and Strep-tagged SHBG were constructed manually by PCR and an In-Fusion HD cloning kit (TaKaRa). Cysteine mutations of MAGT1 and TUSC3 were generated by PCR-directed site mutagenesis. sgRNA sequences specific to *STT3A*, *STT3B*, *MAGT1*, and *TUSC3* were cloned into pSpCas9(BB)-2A-Puro (PX459) according to a standard target sequence cloning protocol described below (75). All of these plasmids were confirmed by Sanger sequencing.

### Virus.

Recombinant LCMV (ARM strain) expressing LASV and MACV GPs (rLCMV/LASV GPC and rLCMV/MACV GPC viruses) were generated by reverse genetic techniques, as previously reported (76, 77). Briefly, we constructed plasmids containing full-length antigenomic cDNA of the LCMV (ARM strain) L and S segments (or an S segment in which NP was linked with GFP via a P2A self-cleaving peptide sequence) flanked by a T7 promoter in their 5′ termini and a hepatitis delta riboenzyme sequence in their 3′ termini, respectively. Then, LCMV GP was replaced by LASV GP (Josiah strain) or MACV GP (Carvallo strain) using PCR and an In-Fusion HD cloning kit (TaKaRa). The two plasmids were cotransfected into T7 RNA polymerase stably expressing cells (BSR-T7) to direct the intracellular synthesis of viral L and S RNA antigenomes and the initial translation of NP and LP. Four days after transfection, the initially rescued viruses were transferred into BHK-21 cells for further amplification.

### Antibodies and reagents.

Antisera specific to viral GP1, GP2, and NP were generated by immunizing BALB/c mice with purified viral proteins. The anti-STT3A (HPA030735) and anti-STT3B (HPA036646) antibodies were purchased from Sigma-Aldrich (USA). The anti-Strep (catalog no. A00626) antibody was purchased from GenScript (China). The anti-V5 (AB3792) antibody was purchased from Merck Millipore (Germany). The anti-MAGT1 (17430-1-AP) and anti-6×His (66005-1-Ig) antibodies were purchased from Proteintech (China). The anti-TUSC3 (A12571), anti-GAPDH (AC002), and anti-Flag (AE005) antibodies were purchased from ABclonal (China). MagStrep XT beads (2-4090-002) and buffer BXT (2-1041-250) were purchased from IBA Life Sciences (Germany). NGI-1 (HY-117383) was purchased from MedChemExpress (China).

### Coimmunoprecipitation/affinity purification and mass spectrometry.

For AP-MS, 5.0 × 10^6^ cells preseeded in 10-cm dishes were transfected with 10 μg of plasmid encoding LASV GP with a C-terminal Twin-Strep-tag or an equal amount of plasmid encoding the single Twin-Strep-tag as a control using the cationic polymer polyethylenimine. For coimmunoprecipitation, plasmids encoding the OST subunits were cotransfected into HEK293T cells with Twin-Strep-tagged LASV GP or GFP as a control bait. At 48 h posttransfection, the cells were collected and washed with prechilled PBS by low-speed centrifugation. The cells were then lysed in 500 μl of prechilled lysis buffer (50 mM Tris, 150 mM NaCl, 5% glycerol, 1% NP-40, and complete protease inhibitor cocktail [Roche]; pH 8.0) for 1 h and cleared by centrifugation at 4°C for 20 min at 13,000 × *g*. The supernatant was incubated with 60 μl of MagStrep XT beads overnight at 4°C. Then, the beads were washed with prechilled wash buffer (50 mM Tris [pH 8.0], 150 mM NaCl, and 0.02% Tween 20) three times. For coimmunoprecipitation, purified bead fractions were eluted by buffer BXT, and eluates were subjected to Western blot analysis.

For AP-MS, an innovative in-bead digestion strategy was used to obtain the highest yield. Briefly, washed beads were resuspended in 100 μl of distilled water and then reduced in 2 mM dithiothreitol (Sigma-Aldrich) at 56°C for 30 min, followed by alkylation in 5 mM iodoacetamide (Sigma-Aldrich) for 30 min at room temperature in the dark. Then, 0.5 μg of sequencing-grade modified trypsin (Promega) was added, and the mix was incubated overnight at 37°C. The resulting peptides were separated from the beads by a magnetic rack and subjected to desalination and concentration by C_18_ bonded Solid Phase Extraction Disks (Empore). Purified peptides were then subjected to electrospray ionization, followed by liquid chromatography-mass spectrometry (LC-MS) detection, as described previously (78). The obtained raw MA spectra were subjected to analysis by ProteinPilot version 5.0 for peptide sequence identification against the Swiss-Prot database (downloaded in 2017), which was set to human species restricted. The threshold value of the false discovery rate (FDR) was set to <0.05.

### GO analysis.

The ID list of host interactors was submitted to PANTHER (http://www.pantherdb.org/). Statistical overrepresentation tests for cellular component, molecular function, and biological process annotations were performed by using a Fisher exact test with an FDR correction. Total genes in the *Home sapiens* database were selected as a reference list. The threshold value of the FDR was set to 0.05. Redundant GO terms were then first eliminated by REVIGO (http://revigo.irb.hr/) based on semantic similarity (47) and further summarized manually. The allowed similarity in REVIGO was set to “Medium (0.7).” A semantic similarity measure of “SimRel” was selected. A subset of the terms with ranked fold enrichment values and the number of mapped proteins was visualized in a scatterplot graph. The top representative functional subsets with highest fold enrichment values are highlighted in the central region and listed in tables. Proteins clustered into functional modules based on highly enriched GO terms were visualized by Cytoscape 3.6.0. Note that not all the interactors were exhibited, and one protein may have been classified into more than one subset.

### Western blotting.

Cells were collected and lysed by radioimmunoprecipitation assay lysis buffer (Beyotime, China) supplemented with protease inhibitor cocktail (Roche). Cell lysates were denatured by adding loading buffer (containing β-mercaptoethanol) followed by heating for 10 min at 100°C. The proteins were then separated on SDS–10% PAGE gels (Bio-Rad) and transferred onto polyvinylidene difluoride membranes by a Trans-Blot Turbo rapid transfer system (Bio-Rad) according to the manufacturer’s instructions. The membranes were blocked in 5% defatted milk (dissolved in Tris-buffered saline [TBS]) for 1 h at room temperature and then incubated with a primary antibody for 1.5 h at room temperature or overnight at 4°C. The membranes were then washed extensively in wash buffer (TBS containing 0.1% Tween 20) three times (for 5 min each time) with agitation and incubated with a horseradish peroxidase (HRP)-conjugated secondary antibody (Proteintech, China) for 1 h at room temperature according to the species source of the primary antibody. The membranes were washed three times in wash buffer and imaged using an enhanced chemiluminescence substrate solution (Millipore, Germany) to visualize the protein bands. GAPDH (glyceraldehyde-3-phosphate dehydrogenase) was utilized as a loading control by stripping the membranes with stripping buffer (Beyotime, China) and reprobing with an anti-GAPDH antibody according to the same procedures.

### Plasmid-based CRISPR-Cas9 engineering of knockout cells.

sgRNA sequences targeting exons of the human *STT3A*, *STT3B*, *MAGT1*, or *TUSC3* genes were designed by using the GeneArt CRISPR search and design tool (https://apps.thermofisher.com/apps/crispr/index.html). Predesigned sequences with the fewest predicted off-target cleavage sites were selected. For *STT3A* and *STT3B*, two target sequences were selected for each gene (*STT3A*-target-1, TATATCTCCCGATCTGTGGC; *STT3A*-target-2, ACAGACATTCCGAATGTCGA; *STT3B*-target-1, GACGACTTGTGCTTGCTCTC; STT3B-target-2, CGTGGATGATGCTTTCGAAG). For *MAGT1* and *TUSC3*, one target sequence was selected (*MAGT1*-target-1, ATAACGGAGTAATTTCTCGG; *TUSC3*-target-1, CGCTCAATCTTCCGAATGAA). A pair of partially cDNA oligonucleotides encoding each sgRNA sequence was synthesized and ligated into a pSpCas9(BB)-2A-Puro plasmid (PX459; Addgene, catalog no. 62988), which bears both the Cas9 gene, and the sgRNA scaffold backbone containing an oligonucleotide cloning site. Construction of the plasmids was performed according to a standard target sequence cloning protocol involving an annealing step and a ligation reaction (75). Briefly, the DNA oligonucleotide pairs encoding the 20-nucleotide sgRNA were 5′ phosphorylated by T4 polynucleotide kinase (NEB) and annealed in a thermocycler with the following parameters: 37°C for 30 min, 95°C for 5 min, and cooling to 25°C at 5°C min^−1^. The DNA oligonucleotides were then ligated into the linearized pSpCas9(BB)-2A-Puro vector using Quick ligase (NEB) at room temperature for 10 min. Finally, the ligated plasmids were transformed into a competent E. coli strain, plated on an LB plate containing 100 μg/ml ampicillin, and incubated overnight at 37°C. The colonies were checked by Sanger sequencing for correct sgRNA insertion. Plasmids encoding Cas9 and each sgRNA were isolated and transfected into HEK293T cells using Lipofectamine 2000 transfection reagent (Invitrogen) to direct genome editing. The transfected cells were selected by incubating with 2 μg/ml puromycin for 72 h, and then isogenic cell lines were isolated by serial dilutions and allowed to expand for 2 to 3 weeks. Genomic DNA from the isogenic cell lines was extracted by using a TIANamp genomic DNA kit (DP304; Tiangen Biotech). PCR amplification of the modified regions was defined by Sanger sequencing using specific genomic cleavage detection primers, and cell lines exhibiting frameshift mutations at the corresponding sites were selected and further confirmed by Western blotting. *MAGT1* and *TUSC3* double-knockout cells (*MAGT1*^–^ *TUSC3*^–^) were constructed by transfecting the *TUSC3*-sgRNA plasmid into *MAGT1*^–^ cells to direct a second editing event. Isogenic cell lines bearing the desired editing outcomes were sequenced and selected in the same way.

### Virus-cell binding assay.

BHK-21 cells were preseeded in 12-well plates in advance and incubated overnight to reach 90 to 100% confluence. Prechilled cells were infected with RNA-normalized viruses derived from *STT3A*^–^, *STT3B*^–^, and WT cells and incubated at 4°C for 60 min. After incubation, the cells were washed extensively with prechilled PBS to remove unbound viral particles. The cellular total RNA was then extracted and purified using a total RNA kit (Omega). Cell-attached viral S genomes were measured by qRT-PCR and normalized to the level of GAPDH mRNA.

### Quantitative reverse transcription-PCR.

RNA was extracted from cells and supernatants with a total RNA kit and a viral RNA kit (Omega) according to the manufacturer’s instructions. Reverse transcription was performed using the PrimeScript RT reagent kit with gDNA Eraser (TaKaRa, Japan) and random primers. Specifically, S genome (+) cDNA of the rLCMV/LASV GPC virus was synthesized using a genome-specific primer (GTACAAGCGCTCACAGACCT) to distinguish from the S antigenome (–) or viral mRNA. qRT-PCR was performed using TB Green Premix Ex Taq II (TaKaRa, Japan) and the following gene-specific primer pairs: S genome (+) (forward, GTACAAGCGCTCACAGACCT; reverse, GTTACCCCCATCCAACAGGG) and GAPDH (forward, AGAAGGCTGGGGCTCATTTG; reverse, AGGGGCCATCCACAGTCTTC). For supernatant viral RNAs, the absolute quantity of the viral S genome was determined by performing a standard curve experiment using an S genome-encoding plasmid as a standard sample. For cellular RNAs, the relative quantity of the viral S genome was determined by performing a comparative *C_T_* (ΔΔ*C_T_*) experiment using GAPDH as an endogenous control.

### Immunological plaque assay.

BHK-21 cells preseeded in 24-well plates were infected with 10-fold serial dilutions of virus. After incubation for 1 h, viral supernatants were discarded, and DMEM containing 1.1% sodium carboxymethylcellulose and 2% FBS was added to the cells. At 48 h postinfection, the cells were fixed with 4% paraformaldehyde for 30 min at room temperature, permeabilized, and blocked in 5% defatted milk containing 0.3% Triton X-100 (dissolved in PBS) for 1 h at room temperature. Next, the cells were incubated with anti-NP serum (1:200 dilution) for 1 h at room temperature or 4°C overnight. Then, the plates were washed extensively in wash buffer (PBS containing 0.05% Tween 20) three times for 5 min each, followed by incubation with an HRP-conjugated anti-mouse secondary antibody (1:300 dilution) for 1 h at room temperature. Finally, the cells were washed three more times, and viral plaques were stained using an enhanced HRP-DAB chromogenic substrate kit (Tiangen Biotech, China). Viral titers were determined based on the number of PFU.

### Immunofluorescence.

Confocal microscopy was used to visualize the cell surface transport of tagged or untagged LASV GP in HEK293T cells. HEK293T cells were seeded in 15-mm glass-bottom culture dishes (801002; NEST Biotechnology) and transfected the next day with pCAGGs plasmids encoding Twin-Strep-tagged or untagged LASV GP using Lipofectamine 2000 transfection reagent (Invitrogen). At 36 h posttransfection, living cells were incubated with a 1:50 dilution of GP2-specific serum in PBS supplemented with 3% FBS at 4°C for 30 min. After three washes in PBS supplemented with 3% FBS, the cells were incubated with a 1:200 dilution of Dylight 488-labeled anti-mouse IgG(H+L) (KPL, catalog no. 072-03-18-06) and washed three times with PBS. After live-cell staining, the cells were fixed in 4% paraformaldehyde, stained with DAPI (4′,6′-diamidino-2-phenylindole) for 10 min, washed three times with PBS, and then subjected to imaging using a Nikon A1 MP multiphoton confocal microscope. Images were captured under a 60× lens objective.

### Ultracentrifugation of viral particles.

Viral supernatants derived from different cells were first cleared by two rounds of centrifugation at 1,000 × *g* and 10,000 × *g* for 30 min to remove cell debris. Cleared supernatants were then ultracentrifuged at 20,000 rpm in a SW32 Ti rotor (Beckman) for 2.5 h at 4°C and layered onto a 20% sucrose cushion. The supernatants were discarded, and the pellets were gently resuspended in PBS and subjected to Western blot analysis.

## Supplementary Material

Supplemental file 1
